# The Effects of Oral Probiotics on Type 2 Diabetes Mellitus (T2DM): A Clinical Trial Systematic Literature Review

**DOI:** 10.3390/nu15214690

**Published:** 2023-11-05

**Authors:** Simon Paquette, Sean C. Thomas, Krishnan Venkataraman, Vasu D. Appanna, Sujeenthar Tharmalingam

**Affiliations:** 1 Medical Sciences Division, NOSM University, Sudbury, ON P3E 2C6, Canada; sipaquette@nosm.ca (S.P.); sethomas@nosm.ca (S.C.T.); kvenkataraman@laurentian.ca (K.V.); 2 School of Natural Sciences, Laurentian University, Sudbury, ON P3E 2C6, Canada; vappanna@laurentian.ca; 3 Health Sciences North Research Institute, Sudbury, ON P3E 2H2, Canada

**Keywords:** probiotic, type 2 diabetes mellitus, T2DM, glucose, gut health, glycemic index, HbA1c, metformin, microbiome, microbiota, lipids

## Abstract

Type 2 diabetes mellitus (T2DM) remains a global health concern. Emerging clinical trial (CT) evidence suggests that probiotic intervention may promote a healthy gut microbiome in individuals with T2DM, thereby improving management of the disease. This systematic literature review summarizes thirty-three CTs investigating the use of oral probiotics for the management of T2DM. Here, twenty-one studies (64%) demonstrated an improvement in at least one glycemic parameter, while fifteen studies (45%) showed an improvement in at least one lipid parameter. However, no article in this review was able to establish a uniform decrease in glycemic, lipid, or blood pressure profiles. The lack of consistency across the studies may be attributed to differences in probiotic composition, duration of probiotic consumption, and probiotic dose. An interesting finding of this literature review was the beneficial trend of metformin and probiotic co-administration. Here, patients with T2DM taking metformin demonstrated enhanced glycemic control via the co-administration of probiotics. Taken together, the overall positive findings reported across the studies in combination with minimal adverse effects constitute ground for further quality CTs. This review provides recommendations for future CTs that may address the shortcomings of the current studies and help to extract useful data from future investigations of the use of probiotics in T2DM management.

## 1. Introduction

Type 2 diabetes mellitus (T2DM) is a chronic metabolic disorder in which the body cannot secrete enough insulin or does not respond appropriately to insulin [1]. Individuals with T2DM have increased blood glucose levels in the absence of adequate insulin action. This hyperglycemia is a common effect of uncontrolled diabetes and over time leads to serious damage to many of the body’s systems, especially the nerves and blood vessels [2]. According to Diabetes Canada, T2DM is caused by several different risk factors and accounts for 90% of diabetes cases in Canada. The International Diabetes Federation predicts that the global prevalence of T2DM would climb from 10.5% in 2021 to 12.2% by 2045 [3].

The risk factors for T2DM are numerous and complex. T2DM develops and progresses due to a combination of genetic, metabolic, and environmental factors. Although T2DM has a genetic predisposition basis, epidemiological research suggests that T2DM can be controlled by adjusting modifiable risk factors that influence its development, such as lifestyle and dietary habits [4]. A growing body of clinical and experimental evidence suggests that dysbiosis in the human gut flora is important in the etiology of T2DM. It is being suggested that an unbalanced gut microbiota is connected to impaired host glycemic regulation and the development of T2DM [5]. Diabetes prevalence, diabetes-related death, and diabetes-related health expenditure continue to climb globally, with significant social, economic, and health-system ramification [3]. Thus, more research is needed to determine and implement new evidence-based advancements for T2DM management.

The human gastrointestinal system is home to the biggest microbial population in the human body, which consists of billions of microbes collectively known as the gut microbiota [6,7]. The normal microbiome performs various physiological roles, including nutrient absorption, host defense and immunity [6,8]. Numerous studies have revealed a bidirectional interaction between gut microbiota and various organs in the human body [6,9]. A large body of research shows that a change in the gut microbiota is a significant component in the etiology of many local and systemic illnesses [6,10]. However, whereas non-human mammalian studies clearly indicate a causal association between gut flora profiles and metabolic syndrome conditions, the relationship in humans remains contentious [11]. Evolving data suggest that gut microbiota composition is an important pathophysiological factor associated with T2DM and has been proposed to help to explain why only certain individuals become burdened by disease [12,13]. A systematic review by Gurung et al. (2020) [12] identified that Bifidobacterium, Bacteroides, Faecalibacterium, Akkermansia, and Roseburia were shown to be negatively associated with T2DM, whereas Ruminococcus, Fusobacterium, and Blautia were found to be favorably associated with T2DM.

Probiotics are commonly defined as live microorganisms which, when administered in adequate amounts, confer a health benefit to the host [14,15]. Prebiotics are commonly defined as non-viable food component that provides health benefit to the host associated with modulation of the microbiota [14]. When administered together, probiotics and prebiotics are referred to as synbiotics and are suggested to synergize together to improve the viability of gut modulation. This review uses the term probiotic rather than synbiotic (unless specified by a publication) as we are interested in the sustained renewal aspect of this treatment. Probiotic therapy is still a relatively novel approach to re-establish a normal gut flora. A healthy gut microbiome is established based on the composition and proportion of various bacteria species [9]. Prebiotics, like inulin and polyphenols, are selectively utilized by the gut microbiome, releasing short-chain fatty acids (SCFAa) and other metabolites which may reduce the intestinal lumen pH, inhibit growth of pathogens, and enhance mineral and vitamin bioavailability [16]. A probiotic microorganism may increase the microbial diversity of the gut microbiome and improve the integrity of the intestinal barrier, leading to an improvement of baseline and pathologic inflammation [16]. As per a review by Gebrayel et al. (2022) [17], there are multiple methods of manipulating the gut microbiota in the prevention and treatment of diseases. Several preclinical studies have found that probiotic administration can lower blood glucose levels and improve various metabolic parameters that affect glycemia. Probiotics, synbiotics, and other gut microbiota modulators may thereby address the regulation and development of T2DM [13]. In sum, these studies suggest that probiotic intervention can promote a healthy gut microbiome in individuals with T2DM, thereby improving management of the condition.

While non-human studies demonstrate encouraging results regarding probiotic intervention for T2DM, human CTs are still emerging. The overall goal of this review article is to provide an overview of CT research that have evaluated the effectiveness of probiotics in the management of T2DM. Specific topics reviewed include glycemic metabolism, lipid profile, inflammation, cardiovascular parameters, and fecal analysis. In addition, this review discusses probiotic species composition and combined pharmacological intervention. Like pharmacological treatment, probiotics may have the potential to improve the quality of life of patients with T2DM. This novel form of therapy is continuously improving and is an important consideration in the arms race for successfully managing T2DM.

## 2. Methods

### 2.1. Data Sources, Search Strategy, and Selection Process

**Figure 1:**
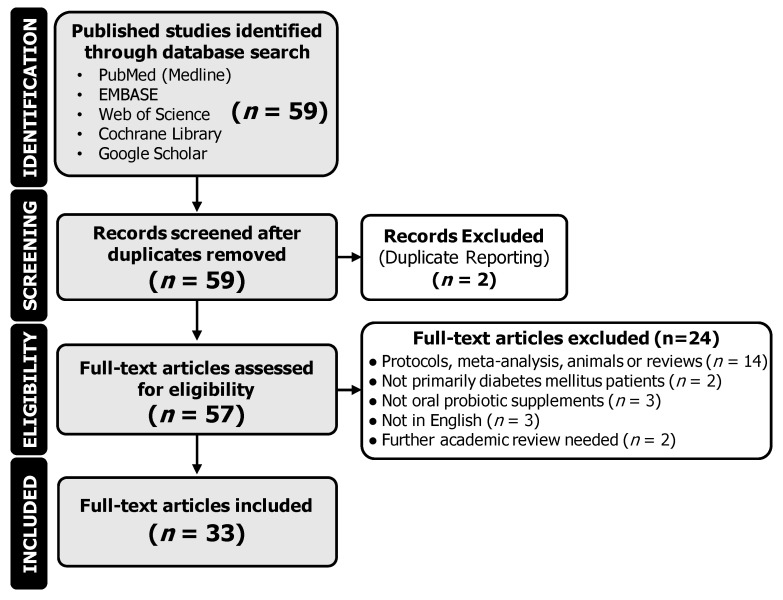
Flow diagram of identified citations and included clinical trials.

The literature search was performed using PubMed (Medline), Google Scholar, EMBASE, Web of Science, and Cochrane Library as previously described using the PRISMA 2020 checklist [18]. Articles published until July 2023 were included. The first author conducted the below-mentioned steps while the last author reviewed and screened all uncertainty flagged by the first author. On PubMed, EMBASE, Web of Science, and Cochrane Library, the advanced search function was used. Titles and abstracts were searched for the keywords “Type 2 Diabetes Mellitus” and “Probiotic*” using the “Add with AND” search function between keywords. The search was then narrowed to “Clinical Trial” and “Randomized Controlled Trial”. On Google Scholar, the ordinary search function was used and searched for the keywords “Type 2 Diabetes Mellitus” and “Probiotic*”. At this stage, 59 unique articles were identified. The trials were then included if they fulfilled the following inclusion/exclusion criteria: (A) the trial was clinical, completed, and included human participants (animal studies, protocols, meta-analyses, and reviews were excluded), (B) participants had T2DM (prediabetic and comorbidity studies were excluded), (C) the effects of orally ingested probiotic supplementation were investigated (non-oral probiotic supplements were excluded), (D) participants were over the age of 18, (E) the article was available in English, and (F) no concern of further academic review was noted. No other restrictions were applied. Upon implementing the eligibility criteria, 24 articles were excluded. This concluded the article search with a total of 35 articles. Of these articles, there were 2 distinct pairs of articles that were from the same data set but split into two articles. This was identified by either identical CT registrations or identical participant characteristics. These articles were, therefore, treated as the same trial, resulting in the inclusion of 33 trials. A PRISMA diagram outlining the search procedure is provided in Figure 1.

### 2.2. Data Collection and Interpretation

The following characteristics were sought in each study: authors, year of publication, DOI, geography (assumed as country of institution if not specified), CT type, number of true participants (met eligibility and started trial), and number of completed participants (finished the trial). In the control and interest groups, the following population characteristics were identified in each study: number of participants, mean age of participants, and SD (±) of age of participants. The following probiotic characteristics were sought in each study: duration of administration (standardized to months), average CFU/dose, frequency/day (assumed 1 if not indicated), average total CFU/day, mechanism/vector, composition, probiotic, placebo substitute, number of different strains, specific probiotic strains, and general genus. The following results were identified in each study: glycemic parameters, lipid parameters, anthropometric measurements, cardiovascular and kidney indicators, inflammation parameters (including markers of oxidative stress), and fecal analysis. An internal assessment was performed for potential biases.

## 3. Results

The results section summarizes the general study characteristics, probiotic composition, biochemical parameters, and fecal microbiome characteristics. The biochemical results from the clinical studies were organized based on the duration of probiotic administration.

### 3.1. Study Characteristics

The characteristics of the 33 CTs extracted from the various databases are summarized in Table 1, presented in chronological order. The total number of participants in the collective studies was 2492, where 2185 persons completed the trials. Trials were conducted in different countries of varying populations with different socioeconomic realities. The countries involved in this review included the following: Australia, Brazil, China, Cuba, Egypt, India, Indonesia, Iran, Japan, Malaysia, Romania, Saudi Arabia, Thailand, and the United States. The country with the most articles was Iran. 

The inclusion criteria of most studies were similar. Generally, participants were within a certain age range, with that range always being in adult populations (minimum over 18 years of age). All participants had T2DM, whether previously diagnosed according to guidelines or by clinical evaluation. Few articles included subjects that had impaired glucose tolerance (such as Palacios et al. (2020) [19]). 

The exclusion criteria of most studies had some similarities. Generally, participants needed to be free of cancer and major diseases such as liver, kidney, and heart diseases. Participants were also generally excluded if they were pregnant, allergic to the trial components, and had taken antibiotics or probiotics within a certain time frame before/during the trial. Pharmacotherapy was quite varied throughout the studies and not well specified, but participants were generally excluded from the studies if they had a change in medication. Several studies excluded participants using insulin.

**Table 1:** Study characteristics.

Article Identification			Trial Characteristics											
Authors, Year of Publication	Citation	DOI (https://doi.org/)	Geography	CT Type	Number of True Participants	Number of Completed Participants	Control Group				Interest Group			
							Placebo or None	Number of Participants	Mean Age	SD (±) of Age	Probiotic	Number of Participants	Mean Age	SD (±) of Age
Chen et al., 2023	[20]	10.1128/msystems.01300-22	China	Two-phase, R, DB	58	48	Placebo	29	46.9	11.25	Probiotic	29	48.7	11.11
García et al., 2023	[21]	10.26502/jbb.2642-91280090	Cuba	R, DB	64	57	Placebo	30	53.2	7.6	Sugar shift (SS) cohort	30	56.3	6.7
Hasanpour et al., 2023	[22]	10.1186/s12902-023-01290-w	Iran	R, DB	100	92	Soymilk + placebo	25	54.24	6.58	Soymilk + Probiotic	25	51.16	7.16
							Milk + placebo	25	52.06	11.42	Probiotic	25	54.4	8.72
Mirjalili et al., 2023	[23]	10.1016/j.clnesp.2023.01.014	Iran	R, DB	72	60	Conventional yogurt	36	58.1	9.8	Probiotic yogurt	36	54.5	8
Ahmadian et al., 2022	[24]	10.1186/s13098-022-00822-z	Iran	R, DB	68	60	Placebo	30	61	Range: 57 to 65	Probiotic	30	58.5	Range: 52 to 64
Gupta et al., 2022	[25]	10.4103/jod.jod_106_21	India	Before-and-after, NB	308	308	N/A	N/A	N/A	N/A	Participants	308	54.2	10.9
Hata et al., 2022	[26]	10.1111/jdi.13698	Japan	Open-label, single-arm, exploratory research	40	36	N/A	N/A	N/A	N/A	Participants	40	64	9.4
Kumar et al., 2022	[27]	N/A	India	R, DB	150	150	Metformin only	75	51.1	5.4	Metformin + probiotic	75	50.9	6.2
Ziegler et al., 2022	[28]	10.1016/j.clnesp.2022.04.002	Brazil	Before-and-after, NB	20	17	N/A	N/A	N/A	N/A	Participants	20	62.5	N/A
Aron et al., 2021	[29]	10.31688/ABMU.2021.56.2.09	Romania	Patient choice, prospective 3-month comparative study, NB	41	41	Control	22	58.14	11.17	Study group	19	60.74	5.84
Ismail et al., 2021	[30]	10.5603/DK.a2021.0037	Egypt	Pilot, NB	150	150	Diet only	50	46.4	13.2	Yogurt and diet	50	48.3	12.9
											Yeast and diet	50	48.6	11.5
Jiang et al., 2021	[31]	10.1002/jcla.23650	China	R, DB, PG	101	76	Placebo	34	56.12	8.23	Probiotic	42	55.96	8.45
Kanazawa et al., 2021	[32]	10.3390/nu13020558	Japan	R, NB	88	80	Control	42	55.9	10.7	Synbiotic	44	61.1	11
Toejing et al., 2021	[33]	10.3390/foods10071455	Thailand	R, DB	50	36	Placebo	18	61.8	7.7	Probiotic	18	63.5	5.9
Palacios et al., 2020	[19]	10.3390/nu12072041	Australia	R, DB	60	53	Placebo	30	56.1	12.3	Probiotic	30	61.4	8.9
Khalili et al., 2019	[34]	10.29252/.23.1.68.	Iran	R, DB, PG	40	NS	Placebo	20	45	5.37	Intervention group	20	43.95	8.14
Lestari et al., 2019	[35]	10.2478/rjdnmd-2019-0041	Indonesia	R, DB	38	32	Control	19	53	10	Intervention	19	56	7
Madempudi et al., 2019	[36]	10.1371/journal.pone.0225168	India	R, DB	79	74	Placebo	39	50.6	N/A	UB0316	40	54.1	N/A
Sabico et al., 2019 Sabico et al., 2017	[37,38]	10.1016/j.clnu.2018.08.009 10.1186/s12967-017-1354-x.	Saudi Arabia	R, DB	96	61	Placebo	39	46.6	5.9	Probiotic	39	48	8.3
Hsieh et al., 2018	[39]	10.1038/s41598-018-35014-1	USA	R, DB	74	68	Placebo	24	55.77	8.55	ADR-1	25	52.32	10.2
											ADR-3	25	53.88	7.78
Raygan et al., 2018	[40]	10.1016/j.pnpbp.2018.02.007	Iran	R, DB	60	52	Placebo	30	67.3	11	Vitamin plus probiotic group	30	71.5	10.9
Feizollahzadeh et al., 2017	[41]	10.1007/s12602-016-9233-y	Iran	R, DB, PG	48	40	Placebo	20	53.6	1.6	Intervention	20	56.9	1.81
Firouzi et al., 2017	[42]	10.1007/s00394-016-1199-8	Malaysia	R, DB, PG	136	101	Placebo	68	54.2	8.3	Probiotic	68	52.9	9.2
Tonucci et al., 2017	[43]	10.1016/j.clnu.2015.11.011	Brazil	R, DB, PG	50	45	Placebo	22	50.95	7.2	Probiotic	23	51.83	6.64
Sato et al., 2017	[44]	10.1038/s41598-017-12535-9	Japan	R, NB	69	68	control	34	65	8.3	Probiotic	34	64	9.2
Asemi et al., 2016	[45]	10.1016/j.clnu.2015.07.009	Iran	R, DB, CO	51	48	Placebo	N/A	N/A	N/A	Beta-carotene fortified synbiotic food group	51	52.9	8.1
Bahmani et al., 2016	[46]	10.1080/07315724.2015.1032443	Iran	R, DB	81	76	Control bread	27	53.4	7.5	synbiotic bread	27	51.3	10.4
Tofighiyan et al., 2016	[47]	10.12691/jfnr-4-12-5	Iran	R, DB	44	42	Placebo	22	54.5	11.1	Probiotic	22	53.45	10.8
Ogawa et al., 2014	[48]	10.1186/1476-511X-13-36	Japan	SB, CO	20	20	Placebo	N/A	N/A	N/A	LG2055 treatment	20	51.1	6.6
Shakeri et al., 2014	[49]	10.1007/s11745-014-3901-z	NS	NS	78	72	Control bread	26	53.1	7.5	Synbiotic bread	26	52.3	10.8
Mohamadshahi et al., 2014	[50]	PMID: 25197295	Iran	R, DB	44	44	Conventional yogurt	22	51	NS	Probiotic yogurt	22	51	NS
Ejtahed et al., 2012 Ejtahed et al., 2011	[51,52]	10.1016/j.nut.2011.08.013 10.3168/jds.2010-4128	Iran	R, DB	64	60	Conventional yogurt	30	51	7.32	Probiotic yogurt	30	50.87	7.68
Moroti et al., 2012	[53]	10.1186/1476-511X-11-29	Brazil	R, DB	50	18	GP (group placebo shake)	9	56.89	1.7	GS (group symbiotic shake)	9	55.47	2

Abbreviations: CO (crossover), DB (double blind), N/A (not applicable), NB (no blinding), NS (not specified), PG (parallel group), R (randomized), and SB (single blind).

The control or placebo groups had a mean number of participants of *n* = 31. These participants had a mean age of 54.2 years with a mean standard deviation of ± 8.1 years, when reported. The probiotic/intervention groups had a mean number of participants of *n* = 32. These participants had a mean age of 54.8 years with a mean standard deviation of ± 8.2 years, when reported. Thus, most of the participants were of middle age. It is important to note that in some cases, intention-to-treat was taken into consideration for the result analysis. The studies that provided information about dropouts stated no serious adverse events related to the probiotics. Most of the observed adverse effects were related to gastric disturbances.

### 3.2. Probiotic Composition

The probiotic characteristics of the 33 clinical studies are summarized in Table 2, presented in chronological order. The average mean duration of probiotic administration was 2.861 months. The range of probiotic administration was of 1 to 6 months (18 studies ≥ 3 months, 15 studies < 3 months). The probiotic composition varied between studies and consisted of the following genera: *Lactobacillus* (twenty-seven studies), *Bifidobacterium* (nineteen studies), *Streptococcus* (seven studies), *Bacillus* (three studies), *Lacticaseibacillus* (two studies), *Saccharomyces* (two studies), *Leuconostoc* (one study), and *Pediococcus* (one study). The diversity of probiotics was further categorized into the following three arbitrary groups: not diverse (one strain), moderately diverse (two to three strains), and very diverse (four or more strains). The results were as follows: not diverse (*n* = 16), moderately diverse (*n* = 7), and very diverse (*n* = 10). One study only used yeast (*Saccharomyces*) as their intervention, and one study used a combination of yeast and bacteria as their intervention. All the other studies in this review exclusively used bacteria as their probiotic component. The most common combination of genera included *Lactobacillus* and *Bifidobacterium* (16 studies). The average mean of the colony forming unit (CFU) per probiotic administration was 1.96 × 10^10^ CFU/dose. The average mean and mode of the frequency of probiotic administration were 1.6 and 1 times/day, respectively. The average mean of the CFU of probiotics administered per day was 3.4 × 10^10^ CFU/day. The mechanism of the oral probiotic delivery varied, with the vectors as follows: bread, capsule, drink, food (not specified), milk, powder, sachet, shake, tablet, and yogurt. When described, the composition and placebo substitute were very diverse. While some trials used proper placebos (i.e., inactive substance, replacing a probiotic component), others used fermented products that contained probiotics.

### 3.3. Biochemical Parameters

A summary of the biochemical results complied from the 33 CTs are presented in Table 3 and Table 4. All trials in this review assessed some kind of blood serum parameter. Glycemic and lipid profiles are by far the most frequently studied. Studies generally focused on the following glycemic indicators: clinical chemistry of glycosylated hemoglobin A1C (HbA1c), fasting plasma glucose (FPG), postprandial glucose (PPG), insulin, and homeostatic model assessment for insulin resistance (HOMA-IR). As for lipid indicators, studies generally focused on the following: total cholesterol (TC), high-density lipoprotein (HDL), low-density lipoprotein (LDL), and triglycerides (TG). Select studies also assessed very low-density lipoprotein (VLDL). Studies generally focused on the following cardiovascular indicators: systolic blood pressure (SBP) and diastolic blood pressure (DBP). In addition, studies reported on the following anthropometric measurements: weight, body mass index (BMI), and waist-to-hip ratio (WHR). However, anthropometric measurements were not always considered as outcomes, and full data were not provided. A few studies also discussed kidney function, inflammation indicators and markers of oxidative stress. All trials used *p* < 0.05 in order to demonstrate the results that were statistically significant. However, the studies interpreted their data in various ways, including the following: trending to significance, significantly different from baseline, significantly different between groups, and significantly different between groups upon addressing covariates.

The data presented in Table 3 and Table 4 highlight significant differences (*p* < 0.05) in the probiotic treatment groups compared to the placebo controls. As shown in Table 3 and Table 4, the 33 CTs were inconsistent in the assessment and reporting of glycemic, lipid, and blood pressure parameters. For example, only 20 studies presented HbA1c levels, while four studies lacked results for all glycemic parameters. Therefore, selective reporting of biochemical parameters was identified as the primary bias across the studies. Many studies also failed to disclose information on the antidiabetic medications utilized by the subjects. No other biases were apparent from our review. 

The text below summarizes the key biochemical and anthropometric findings of each study organized into two groups: probiotic intervention for ≥ 3 months (Table 3) and probiotic intervention for < 3 months (Table 4). In certain studies, the probiotic composition included a prebiotic. The composition of the prebiotic is listed in Table 3 and Table 4 when relevant, in addition to the duration of probiotic intervention.

**Probiotics Intervention for ≥ 3 Months (Table 3)**

Chen et al. (2023) [20] performed a two-phase double-blind randomized control trial where the use of probiotic supplementation with metformin was studied for 3 months. Their results showed that the co-administration of probiotics with metformin significantly reduced HbA1c and homeostasis model assessment-β (HOMA-β) compared to metformin taken alone. The other glycemic indicators such as area under the curve of blood sugar, area under the curve of insulin, HOMA-IR, quantitative insulin sensitivity check index (QUICKI), and Gutt index (insulin sensitivity index, ISI 0,120) did not show significant changes within groups. The lipid indicators such as TC, HDL, LDL, and TG levels did not show significant changes within groups. Metagenomic and metabolomic analyses showed that the co-administration of probiotics increased the abundance of gut SCFA-producing bacteria and bile acids. Significantly or marginally more bile acids and related metabolites were detected in the probiotic group than in the placebo group after intervention. This study showed that the co-administration of probiotics with metformin synergized the hypoglycemic effect in patients with T2DM, which was likely through modulating the gut microbiome. 

These findings were corroborated by Kumar et al. (2022) [27]. Here, probiotic and metformin co-administration for 3 months significantly reduced HBA1c in T2DM subjects compared to patients taking metformin alone. However, the other glycemic and lipid parameters showed no significant differences. Gupta et al. (2022) [25] and Madempudi et al. (2019) [36] also looked at the effects of the probiotic co-administered with stable metformin therapy for 3 months. Gupta et al. (2022) [25] demonstrated improvements in HBA1c, FPG, PPG, TC, LDL, and TG with probiotic administration. Similarly, Madempudi et al. (2019) [36] showed significantly reduced HbA1c and weight with probiotic consumption compared to placebo. However, the changes recorded in FBG, HOMA-IR, insulin, TC, TG, HDL, and LDL levels were not significantly altered. 

Palacios et al. (2020) [19] investigated a highly diverse probiotic (*L. plantarum* Lp-115, *L. bulgaricus* Lb-64, *L. gasseri* Lg-36, *B. breve* Bb-03, *B. animalis* sbsp. *lactis* Bi-07, *B. bifidum* Bb-06, *S. thermophilus* St-21 and *S. boulardii* DBVPG 6763) on adults with T2DM for 3 months. Their results showed no significant differences in FPG, HbA1c, HOMA-IR, fasting plasma insulin (FPI), insulin sensitivity index (Matsuda), anthropometric measurements, lipids, blood pressure, and inflammatory markers. However, an analysis of a subgroup of participants taking metformin showed a decrease in FPG, HbA1c, insulin resistance, and zonulin. Moreover, the probiotic group showed an increase in plasma butyrate concentrations and an enrichment of microbial butyrate-producing pathways in the probiotic group but not in the placebo group.

Firouzi et al. (2017) [42] assessed the effect of a multi-strain probiotic using a 3-month RCT. They found that HbA1c and fasting insulin significantly improved in the probiotic group compared to the placebo group. The following parameters were considered not statistically different: FBG, HOMA-IR, QUICKI, hs-CRP, weight, BMI, waist circumference, TG, TC, HDL, LDL, SBP, and DBP. Also, quantities of *Lactobacillus* spp. and *Bifidobacterium* spp. were assessed and demonstrated a significant increase between the groups, indicating successful probiotic alteration in the gastrointestinal tract.

In a 3-month RCT, moderately diverse probiotic yogurt or unenriched probiotic yogurt was taken twice a day. After adjusting for baseline values of covariate, Mirjalili et al. (2023) [23] found a significant reduction in HbA1c, TC and LDL in the interest group compared to the placebo group. TG, FPG and HDL remained unchanged.

Jiang et al. (2021) [31] looked at the effects of probiotic administration on glycemic control and renal function in patients with diabetic nephropathy using a placebo-controlled RCT. A moderately diverse probiotic was administered via capsule for 3 months and revealed significant reductions in mAlb/Cr, while FBG, HbA1c, PPG, and eGFR remained insignificant in a between-group analysis.

In another 3-month double-blind RCT by García et al. (2023) [21], participants took a probiotic capsule with multiple strains or a placebo. The interest group demonstrated stabilization of their FBG, PPG and TC levels as those levels in the placebo group significantly increased. Insulin levels, HOMA-IR and serum LPS decreased significantly in the interest group compared to placebo. The HbA1c, HDL, LDL, TG, and creatinine levels did not show significant changes between the groups.

Ziegler et al. (2022) [28] conducted a pilot CT where 20 patients who were overweight or obese and had T2DM were assessed before and after intervention. The patients drank a probiotic of *Bacillus clausii* spores daily for 3 months. Though lack of control and blinding, the results showed statistically significant difference in blood glucose, increased HDL plasma levels, and improved intestinal microbiota profiles (assessed via *E. coli* growth patterns) among the patients included in this study. TC, TG, LDL, VLDL, non-HDL cholesterol, BMI, SBP, and DBP remained unchanged. 

Toejing et al. (2022) [33] illustrated the efficacy of *L. paracasei* on glycemia in T2DM patients. Compared to controls, *L. paracasei* administration improved FPG, LDL and HDL levels compared to placebo controls. However, this study failed to show improvements in HBA1c, TC and TG. 

Aron et al. (2021) [29] conducted a trial where subjects who had metabolic syndrome (MS) and T2DM chose the treatment option: no modification or probiotics. The probiotics were individualized for the participants, and all contained *Bacillus* spores. A group comparison showed insignificant changes to BG, HbA1c, TC, TG, HDL, LDL, BMI, SBP, and DBP. However, weight loss was significantly higher in the study group compared to the control group. 

Raygan et al. (2018) [40] studied the effects of vitamin D with probiotic supplementation on patients with T2DM with chronic heart disease. Subjects were randomly allocated into two groups that received either 50,000 IU vitamin D every 2 weeks plus a daily diverse probiotic or placebo for 3 months. Compared to the placebo, the vitamin D and probiotic co-supplementation resulted in significant improvements in beck depression inventory total score, beck anxiety inventory scores, and general health questionnaire scores as well as significant reductions in serum insulin HOMA-IR, and serum 25-OH-vitamin D, the quantitative insulin sensitivity check index, serum HDL-cholesterol levels, C-reactive protein (CRP), plasma nitric oxide (NO), and plasma total antioxidant capacity (TAC). However, no significant changes were seen in FPG, TG, TC, VLDL, LDL, total glutathione (GSH), malondialdehyde (MDA), SBP and DBP.

Ismail et al. (2021) [30] completed a 4-month CT where they had three groups partake in a well-balanced diet: the control group only had the dietary change, whereas the two interest groups either had two cups of yogurt daily or had one teaspoonful of yeast daily. All three groups showed a significant reduction in PPG levels and LDL, and thus their reduction cannot be attributed to probiotic supplementation alone. The patients receiving yogurt and the patients receiving yeast showed a significant reduction in FBG, HbA1c, IL6, TNF-a, and CRP and a significant increase in HDL compared to the patients on the diet change only.

Sato et al. (2017) [44] performed an interventional RCT to investigate whether probiotics could reduce bacterial translocation and cause changes in the gut microbiota. The trial was conducted for 4 months, where one group drank probiotic milk, and the other was a control. Upon completion of the trial, there was no significant difference between BMI, HbA1c, glycoalbumin, FBG, fasting C-peptide, TC, HDL, TG, hs-CRP, TNF-α, IL-6, adiponectin, and LBP. At the end of the study, the fecal counts of the *Clostridium coccoides* group and *Clostridium leptum* subgroup in the probiotic group were significantly higher than in the control group. As expected, the fecal counts of total *Lactobacillus* were significantly higher in the probiotic group. Intriguingly, the total count of blood bacteria was significantly lower in the probiotic group. However, fecal organic acids were comparable between the two groups.

Sabico et al. (2017/2019) [37,38] looked at the effects of a highly diverse probiotic. The following parameters were evaluated: glucose, insulin, C-peptide, HOMA-IR, TG, TC, HDL, LDL, TNF alpha, IL-6, CRP, leptin, adiponectin, resistin, endotoxin, BMI, WHR, SBP, DBP, and MAP. Only HOMA-IR had a clinically significant reduction in the probiotic group as compared to the placebo group at all time points. No other clinically significant changes were observed between the probiotic and placebo groups at 3 and 6 months in other markers.

Kanazawa et al. (2021) [32] investigated the effects of a moderately diverse synbiotic supplementation on chronic inflammation and the gut microbiota in patients with obesity and T2DM during a 6-month period. Although no significant changes in inflammatory markers were found by a between-group comparison, synbiotic administration at least partially improved the gut environment in obese patients with T2DM. 

Hsieh et al. (2018) [39] first identified the antidiabetic effects of *L. reuteri* strain ADR-1 on a rat model. They then conducted a 6-month RCT with three groups: *L. reuteri* strain ADR-1, *L. reuteri* strain ADR-3 (heat-killed) with placebo. The following parameters were evaluated in the study: HbA1c, insulin, HOMA-IR, glucose AC, LDL, HDL, TG, TC, FFA, SBP, DBP, pulse pressure, mean pressure, C-peptide, CRP, IL-6, IL-10, IL-17, TNF-α, IL-1β, SOD, and GPX. When compared to placebo, *L. reuteri* strain ADR-1 showed significant reductions in TC after 3 months (but not 6 months) and HbA1c after 3 and 6 months. When compared to placebo, *L. reuteri* strain ADR-3 (heat-killed) showed a significant decrease in SBP, mean pressure, and IL-1β after 6 months. All other parameters were insignificant. The analysis of fecal microflora found that *L. reuteri* were significantly increased in the ADR-1 group and *Bifidobacterium* spp. were significantly increased in the *L. reuteri* strain ADR-3 group after 6 months. 

In summary, 15 out of 18 studies (83%) that investigated the use of probiotic administration for ≥ 3 months demonstrated an improvement in at least one glycemic parameter in patients with T2DM. In particular, 10 out of 15 studies (67%) showed a significant reduction in HbA1c. Interestingly, all four CTs that specified a study population consisting of T2DM patients on metformin exhibited a significant reduction in HbA1c. In regard to lipid assessment, seven out of seventeen studies (41%) with ≥ 3 months of probiotic intervention exhibited an improvement in at least one lipid parameter, while one out of five studies (20%) exhibited an improvement in blood pressure regulation. However, there were no studies that showed consistent change in all glycemic, lipid, or blood pressure parameters. In addition, it is important to note that 10 out of 18 studies (56%) with ≥ 3 months intervention utilized probiotics consisting of *Lactobacillus* and *Bifidobacterium* genera.

**Probiotics Intervention for < 3 Months (Table 4)**

Hata et al. (2022) [26] conducted an open-label, single-arm exploratory study where patients treated with metformin were given probiotic BBG9-1 for 2.5 months. They found that the gastrointestinal symptom rating scale total score significantly improved. However, the FPG and HbA1c levels did not change. The relative abundance of the genus *Sutterella* decreased in the interest group.

Khalili et al. (2019) [34] conducted a trial to evaluate the effect of *L. casei* on glycemic control, serum sirtuin1 (SIRT1), and fetuin-A in patients with T2DM. The participants took a capsule once daily for 2 months. Anthropometric measurements, dietary intake questionnaires, and blood samples were collected, and the patients were assessed by an endocrinologist at the beginning and at the end of the trial. FBG, insulin, and HOMA-IR significantly decreased in the probiotic group compared to the placebo group. However, the HbA1c reduction was not significant. In comparison with placebo, *L. casei* supplementation significantly increased SIRT1 and decreased fetuin-A levels suggesting a novel mechanism of probiotic action in T2DM management. 

Ahmadian et al. (2022) [24] evaluated the effects of probiotic supplementation on CVD-related parameters. Participants were taking capsules that were moderately diverse in probiotics or a placebo for a period of 1.5 months. When compared to the placebo, the probiotic supplementation resulted in a significant decrease in SBP, DBP, mean arterial blood pressure (MAP), the Framingham risk categories, and a log TG/HDL ratio. No significant changes were observed in heart rate (HR), TC/HDL, LDL/HDL, total antioxidant capacity (TAC), paraoxonase (PON), and total oxidant status (TOS). 

Tofighiyan et al. (2016) [47] conducted a 2-month RCT where daily probiotic and placebo tablets were evaluated. The between-group analysis revealed that the probiotic intake caused a significant decrease in serum TC but not in LDL, HDL and TG in the probiotic group.

Hasanpour et al. (2023) [22] looked at the effects of soymilk, conventional milk and probiotic supplements for 1.5 months. Due to multiple interest groups and lack of placebo, between-group comparisons were insignificant. The study claimed that the probiotic supplement significantly reduced the SBP levels compared to conventional milk, but further research is needed to confirm the results. Similarly, Moroti et al. (2012) [53] evaluated the effect of a moderately diverse symbiotic shake consumption on lipid profile in a female T2DM population. Although the study identified trends, the between-group comparison was not completed, and over half of the true participants failed to complete the study.

Ejtahed et al. (2011/2012) [51,52] conducted a 1.5-month RCT. The interest group consumed moderately diverse enriched probiotic yogurt, and those in the control group consumed conventional yogurt. The following parameters were evaluated: glucose, HbA1c, insulin, superoxide dismutase (SOD), glutathione peroxidase (GPx), catalase (CAT), total antioxidant status (TAS), malondialdehyde (MDA), TC, TG, HDL, and LDL. Compared to the control group, the probiotic yogurt significantly decreased FBG, HbA1c, TC, and LDL and increased erythrocyte superoxide dismutase, glutathione peroxidase activities, and total antioxidant status. In addition, the serum MDA concentration significantly decreased compared to the baseline value in both groups.

Lestari et al. (2019) [35] and Mohamadshahi et al. (2014) [50] studied the effects of conventional yogurt to a probiotic-enhanced version for one and two months, respectively. Lestari et al. (2019) [35] evaluated anthropometric indices, dietary intake, physical activity, serum FBG, and lipid profile at the beginning and at the end of the intervention. Their results suggest that the consumption of conventional yogurt significantly reduced FBG levels, whereas probiotic yogurt did not alter FBG levels. Although the TC and TG were not improved after yogurt consumption, both types of yogurt improved HDL levels. Similarly, Mohamadshahi et al. (2014) [50] showed that the probiotic yogurt improved HDL and LDL compared to conventional yogurt. Taken together, both studies concluded that the probiotic yogurt could be used as functional food due to improvements in HDL levels in patients with T2DM. 

Ogawa et al. (2014) [48] conducted a single-blind, placebo-controlled, within-subject, repeated-measure intervention trial where fermented milk with and without a single probiotic was examined for 1 month (with a 1-month washout period, despite the placebo being first for all). The following parameters were evaluated after the intake of oral fat-loading test (OFLT) meals: weight, BMI, waist, TG, nonesterified fatty acid (NEFA), Apo B-48, TC, LDL, HDL, glucose, insulin, amylase, HbA1c, total protein, alanine aminotransferase (ALT), aspartate aminotransferase (AST), alkaline phosphatase (ALP), lactase dehydrogenase (LDH), gamma-glutamyl transpeptidase (γ-GTP), and total bilirubin. The OFLT showed that the postprandial serum NEFA levels were significantly lower than those in the control FM period, and HbA1c was significantly higher.

Feizollahzadeh et al. (2017) [41] evaluated the effects of probiotic soy milk containing *Lactobacillus planetarum* A7 using a 2-month RCT where the interest group consumed probiotic-enriched soy milk, and the control group consumed soy milk. The between-group comparison found that LDL and HDL statistically decreased, while no difference was seen in adiponectin, TNF-a, CRP, FBG, and TG.

Tonucci et al. (2017) [43] looked at the effects of fermented milk for 1.5 months by examining two groups: one with conventional fermented milk and another with enhanced fermented milk. There was a significant difference between the groups concerning the mean changes in HbA1c, TC and LDL. However, the intervention had no significant effect on FPG, fructosamine, insulin, HOMA-IR, TC, HDL-C, TG, total antioxidant status (TAS), and F2-isoprostane. Generally, similar trends were seen by the groups regarding anti-inflammatory markers, SCFA analysis, and markers of oxidative stress; however, none were clinically significant.

Bahmani et al. (2016) [46] and Shakeri et al. (2014) [49] examined the effects of consuming probiotic bread three times daily using a 2-month RCT. Participants were divided into three groups: control bread, probiotic bread containing *Lactobacillus sporogenes*, and synbiotic bread containing *Lactobacillus sporogenes* with inulin. They found that the consumption of the synbiotic bread compared to the probiotic bread and control bread resulted in a significant rise in HDL and plasma NO and a significant reduction in MDA, TG and VLDL levels. There was no significant effect of the synbiotic bread consumption on plasma total antioxidant capacity (TAC), plasma glutathione (GSH), FPG, TC, LDL, catalase, serum liver enzymes, calcium, iron, magnesium levels, weight, BMI, SBP and DBP compared to those for the probiotic bread and control bread.

Asemi et al. (2016) [45] studied the effects of beta-carotene fortified synbiotic food intake through a crossover RCT. Participants consumed either control food or beta-carotene fortified synbiotic food containing *L. sporogenes* and inulin three times a day for 1.5 months. After a 3-week washout, the participants were switched to the opposite group, and the trial was repeated. The consumption of beta-carotene fortified synbiotic food resulted in a significant decrease in insulin, HOMA-IR, HOMA-B, TG, VLDL, and the HDL-TC ratio compared to the control food. In addition, beta-carotene fortified synbiotic food consumption led to elevated plasma NO, glutathione (GSH), and magnesium. However, BMI, weight, FPG, QUICKI, TC, LDL, HDL, CRP, TAC, malondialdehyde (MDA), alanine aminotransferase (ALT), aspartate aminotransferase (AST), alkaline phosphatase (ALP), calcium, iron, SBP and DBP were clinically insignificant.

In summary, six out of ten studies (60%) that investigated the use of probiotic administration for < 3 months demonstrated an improvement in at least one glycemic parameter in patients with T2DM. In particular, two out of five studies (40%) showed a significant reduction in HbA1c. Interestingly, prominent lipid changes were identified in studies that assessed < 3 months of probiotic intervention. Here, eight out of ten studies (80%) exhibited an improvement in at least one lipid parameter. In addition, two out of four studies (50%) exhibited an improvement in blood pressure regulation. However, there were no studies that showed consistent change in all glycemic, lipid, or blood pressure parameters. In addition, it is important to note that eight out of fifteen studies (53%) with < 3 months of intervention utilized probiotics consisting of a single genus (*Lactobacillus* or *Bifidobacterium*).

### 3.4. Fecal Analysis

Several studies looked at the fecal analysis where the parameters and tests differed based on the study. Baseline and end of trial were mostly used for comparison, but few trials also collected fecal samples at other time points. Ziegler et al. (2022) [28] demonstrated that using *B. clausii* supplements reduced *E. coli* growth in patients with T2DM, indicating a promising modulation. Hsieh et al. (2018) [39] investigated *L. reuteri* strains ADR-1 (live) and ADR-3 (heat-killed) for 6 months. When analyzing fecal samples, they found that *L. reuteri* were significantly increased in the ADR-1 consumption group, and *Bifidobacterium* spp. were significantly increased in the ADR-3 consumption group. Sato et al. (2017) [44] found that fecal counts of the *Clostridium coccoides* group and *Clostridium leptum* subgroup in the probiotic group were significantly higher than in the control group at the completion of the study. As expected, the fecal counts of total *Lactobacillus* were significantly higher in the probiotic group, but the total count of blood bacteria was significantly lower in the probiotic group. Nevertheless, fecal organic acids were comparable between the two groups. Kanazawa et al. (2021) [32] found that, relative to baseline, at 24 weeks after synbiotic administration there were positive changes in the counts of *Bifidobacterium*, total *Lactobacilli*, and the concentrations of acetic and butyric acids in feces. They state that synbiotic administration partially improved the gut environment in obese patients with T2DM. Hata et al. (2022) [26] stated that all of their participants were on at least metformin and found no alterations in diversity. However, they did find an increase in the relative abundance of the phylum *Firmicutes* and a relative decrease in the phylum *Bacteroidetes* and the genus of *Sutterella*. Similarly, Palacios et al. (2020) [19] investigated probiotic use in individuals with prediabetes or early T2DM. An analysis of a subgroup of participants taking metformin showed an increase in plasma butyrate concentrations and an enrichment of microbial butyrate-producing pathways in the probiotic group but not in the placebo group. Likewise, Chen et al. (2023) [20] used a diverse probiotic on individuals undertaking metformin therapy. Metagenomic and metabolomic analyses showed that the co-administration of probiotics increased the abundance of gut SCFA-producing bacteria and bile acids. Significantly or marginally more bile acids and related metabolites were detected in the interest group than in the placebo group after intervention.

## 4. Discussion

**Figure 2:**
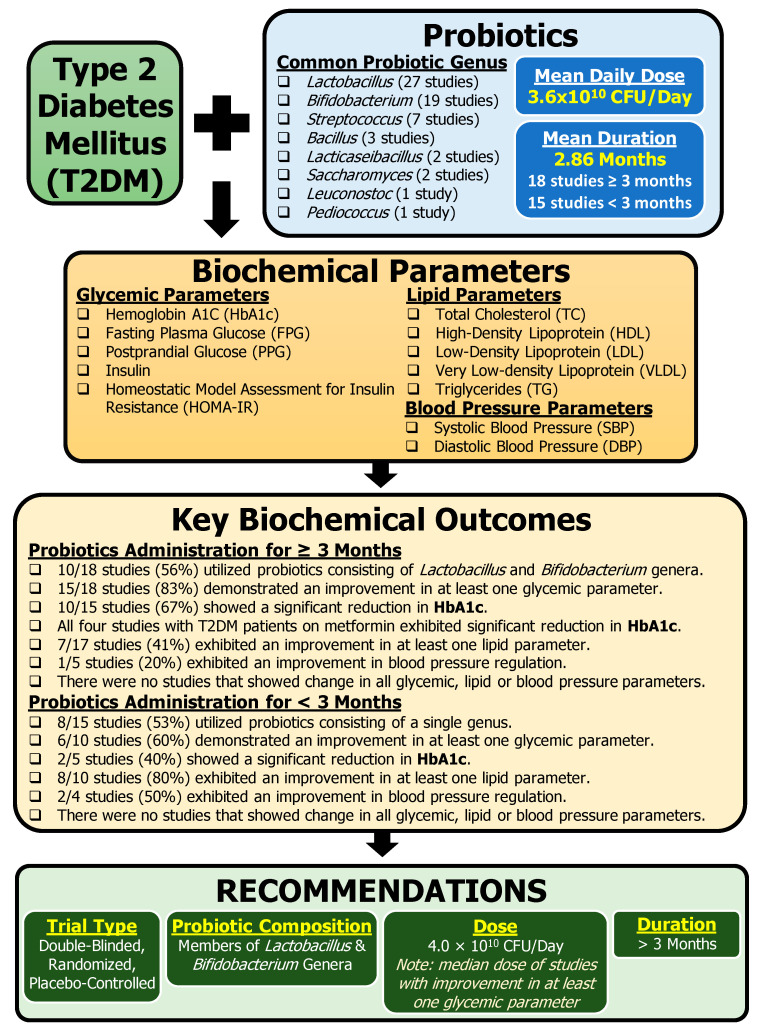
Summary of key information identified from clinical trials assessing the effects of probiotics on T2DM. Based on the information summarized in this review, several recommendations are proposed to address the shortcomings of the current studies and help to extract useful data for future CTs investigating the use of probiotics in T2DM management.

This literature review summarized 33 clinical studies that have investigated the effects of probiotics in the management of T2DM. The Discussion section elaborates on the following themes: glycemic profile, lipid profile, inflammation, cardiovascular and kidney parameters, and metformin use. This section also discusses the limitations and issues of the studies as well as recommendations for future CTs. Key information from this review is summarized in Figure 2.

### 4.1. Glycemic Profile

Probiotic strains may have a positive impact on glycemic control in individuals with T2DM, potentially leading to improved glycemic profile. This review presented 28 studies that assessed one or more glycemic parameter (Table 3 and Table 4). Here, 21 studies (75%) demonstrated an improvement in at least one glycemic parameter in patients with T2DM with probiotic intervention. In particular, 12 studies (57%) showed a significant reduction in HbA1c, a marker of average blood glucose level within the past 2–3 months. However, none of the CTs were able to demonstrate a uniform decrease in all main glycemic profile parameters with probiotic use. Zhang et al. (2022) [54] conducted a review of RCTs to quantify the effect of probiotic administration on glycemic homeostasis in T2DM. They found that, when compared to the placebo, probiotic supplementation did not lead to clinically significant reductions in the HbA1c levels, but the reductions in FPG and fasting insulin levels were of marginal clinical significance [54]. Conversely, a meta-analysis by Ding et al. (2021) [55] supports the notion that probiotic supplementation improved glycemic control, as evidenced by a significant reduction in HbA1c, FPG, and HOMA-IR [55]. The lack of consistency with changes in glycemic parameters with probiotic intervention in T2DM management may be attributed to differences in probiotic composition, duration of probiotic consumption, and probiotic dose.

While HbA1c is a good indicator of average glycemic levels in the past 3 months, the use of HbA1c as an indicator of success for certain CTs is limited, as the average length of probiotic administration was below 3 months across the studies. This may explain why 10 out of the 15 studies that investigated probiotic intervention for < 3 months did not assess HbA1c levels. Moreover, probiotic induced shifts in the gut microbiome composition takes time to adjust [56]. In fact, the time needed to see the beneficial effects of probiotics in patients with T2DM is still not understood. Given that ≥ 3 months of probiotic intervention resulted in higher frequency of studies demonstrating improved glycemic parameters (83% studies compared to 60% studies with < 3 months of intervention), a minimum of 3 months of probiotic administration is likely required for successful glycemic improvements with probiotic intervention for T2DM management. 

In regard to studies that demonstrated nonsignificant changes with probiotic administration, an important factor to consider is the disease progression of participants with T2DM. It is possible that the study subjects might be getting worse as the trials move forward. Here, unchanged glycemic parameters similar to baseline could indicate clinical success. This was the case in the study of García et al. (2023) [21] where the probiotic subjects demonstrated stabilization of their FBG, PPG, and TC levels, while those levels in the placebo group continued to significantly increase. Similarly, the meta-analysis by Zhang et al. (2022) [54] found that the reduction in fasting insulin levels became more significant with the increase in the participants’ age and baseline BMI. A meta-analysis by Naseri et al. (2022) [57] found that weight and BMI were only reduced after synbiotic (not just probiotic) supplementation in individuals with T2DM [58]. Zhang et al. (2022) [54] also found that, when compared to single-strain and low-dose probiotics, multi-strain and high-dose probiotics have a greater beneficial effect on glycemic hemostasis [54]. 

Across the 33 CTs presented in this review, 16 studies (48%) utilized a combination of strains from the *Lactobacillus* and *Bifidobacterium* genera. All 16 studies showed an improvement in at least one glycemic parameter. The positive findings related to these genera might be associated with their respective roles in gut health. For example, members of the *Lactobacillus* genus are known to enhance intestinal barrier defense by promoting mucus secretion [59], while members of the *Bifidobacterium* genus promote digestion of fibers, prevent infections, and enhances production of vitamins and healthy fatty acids [60].

### 4.2. Lipid Profile

Probiotic supplementation may have beneficial effects on lipid parameters for individuals with T2DM. This review presented 27 studies that assessed one or more lipid parameter (Table 3 and Table 4). Here, 15 studies (56%) demonstrated an improvement in at least one lipid parameter in patients with T2DM with probiotic intervention. However, no article in this review was able to demonstrate a uniform decrease in lipid profile parameters. Though clinical significances were achieved in some parameters, to the best of our abilities, these parameters did not follow a trend.

A meta-analysis by Wang et al. (2020) [61] clarified the effect of probiotic intake on dyslipidemia in patients with T2DM. They found that probiotic intake significantly reduced TC and TG but did not alter LDL or HDL concentrations. A subgroup analysis showed that multispecies probiotics (≥two species) rather than single-species probiotics seemed to be the reason. They also found that powder, but not liquid probiotics, could reduce TC and TG concentrations [61]. A meta-analysis by Naseri et al. (2022) [58] investigated the impact of probiotic and synbiotic supplements on health factors in patients with prediabetes and T2DM. With the probiotic/synbiotic intervention, several parameters changed significantly with moderate quality of evidence, including TG, TC, LDL and HDL [58].

Of the trials in this review, none demonstrated simultaneous beneficial effects on TC and TG. Overall, the inconsistency in the findings across the reviewed studies makes it difficult to identify any patterns that support positive effects on lipid profile with probiotic use in patients with T2DM.

### 4.3. Cardiovascular and Kidney Parameters

Ahmadian et al. (2022) [24], Hasanpour et al. (2023 [22]), and Hsieh et al. (2018) [39] reported clinically significant findings regarding the improvement of SBP with probiotic use (Table 3); however, numerous other studies contradict this result. DBP was only cited to be clinically significant by Ahmadian et al. (2022) [24], while no other studies reported this result. The MAP was reported only a few times with the results contradicting each other. Interestingly, the articles citing improvements in SBP, DBP and MAP all used capsules as their probiotic source. Similarly, a meta-analysis by Naseri et al. (2022) [58] investigated the impact of probiotic and synbiotic supplements on cardiovascular health factors in patients with prediabetes and T2DM. With the probiotic/synbiotic intervention, several parameters changed significantly, including SBP and DBP, both being identified as moderate for quality of evidence [58]. 

### 4.4. Inflammation

Probiotics are also being studied for their potential anti-inflammatory and antioxidant properties. Although this review did not assess inflammation as a primary endpoint, select studies provided relevant information regarding the role of inflammatory biomarkers in the pathogenesis of T2DM. Overall, these studies showed that probiotics may help to decrease systemic inflammation and oxidative stress, which are associated with complications in T2DM [6]. Inflammation may be produced by gut barrier abnormalities, SCFA metabolism, and bile acid metabolism as these have been linked to mediate interactions between gut microbiota and host metabolism [11]. Generally, such dysbiosis is directly associated with the development of several illnesses, due to the potential increase in intestinal permeability leading to a systemic inflammation triggered by higher levels of circulating lipopolysaccharides and changes in the immune response caused by an overgrowth of a specific genus of pathogens [16].

García et al. (2023) identified a clinically significant decrease in serum LPS in the probiotic group compared to the placebo group. Ismail et al. (2021) [30] found that participants receiving yogurt and the participants receiving yeast did show a significant reduction in IL-6, TNF-a, and CRP, but Kanazawa et al. (2021) [32], Palacios et al. (2020) [19], Hsieh et al. (2018) [39], Sabico et al. (2017/2019) [37,38], Sato et al. (2017) [44], Bahmani et al. (2016) [46], and Shakeri et al. (2014) [49] demonstrated a differing perspective from this assertion. Other markers including leptin, adiponectin, resistin, and endotoxin were discussed in some trials, though their results were mostly clinically insignificant. Interestingly, Hsieh et al. (2018) [39] found that IL-1β levels improved in only a heat killed *L. reuteri* but not live. Bahmani et al. (2016) [46] found that the consumption of synbiotic bread compared to probiotic bread and control bread resulted in a significant reduction in malondialdehyde (MDA) levels. As for other oxidative stress markers, Ahmadian et al. (2022) [24], Bahmani et al. (2016) [46], and Shakeri et al. (2014) [49] looked at many markers, but their results also showed no trends. 

A meta-analysis by Ding et al. (2021) [55] investigated the effects of probiotics on inflammatory markers in adults with T2DM. They demonstrated that, compared to the control group, probiotic intake produced a beneficial effect in reducing the levels of plasma inflammation markers, including TNF-α and CRP, while it had no effect on IL-6 (Ding et al., 2021) [55]. A meta-analysis by Naseri et al. (2023) [57] investigated the probiotic or synbiotic intake on adipokines, inflammation, and oxidative stress in patients with prediabetes and T2DM. Their results support the notion that probiotic or synbiotic intake in individuals with T2DM may reduce CRP, TNF-α, and MDA and increase TAC and GSH but have no effects on IL-6, adiponectin, and leptin when compared to the control group (Naseri et al., 2023) [57]. Though this meta-analysis looked at slightly different parameters and factors compared to this review, their results illustrate possible benefits of probiotics with reducing inflammation. However, the claims regarding inflammation from the results in this review are few and contradictory. With the studies reviewed in this article, the effects of probiotics on inflammation in patients with T2DM remain indecisive.

### 4.5. Metformin and Probiotics as Adjunctive Therapy

An interesting finding in this literature review was the beneficial trend of metformin and probiotic co-administration. This finding was of interest clinically as metformin and lifestyle changes are the gold standard treatment for T2DM [62]. 

Palacios et al. (2020) [19] performed a subgroup analysis of their participants taking metformin. In this specific population, they found a significant decrease in FPG, HbA1c, insulin resistance, and zonulin. Moreover, the probiotic group showed an increase in plasma butyrate concentrations and an enrichment of microbial butyrate-producing pathways in the probiotic group but not in the placebo group. Madempudi et al. (2019) [36] and Kumar et al. (2022) [27] also identified significantly reduced HbA1c with probiotic use in patients with T2DM taking metformin. 

More recent studies assessed the effects of probiotics and metformin therapy on the gut microbiome composition. For example, Hata et al. (2022) [26] examined the alternation in abundance of the microbiome population with probiotic consumption in patients with T2DM on metformin. Here, there was an increase in the relative abundance of the phylum *Firmicutes*, a decrease in the phylum *Bacteroidetes,* and a decrease in the genus *Sutterella*. It has been reported that *Sutterella* species are involved in mild pro-inflammatory activity [63]. They also found that the *gastrointestinal symptom rating scale* score significantly improved in their probiotic group. This finding was clinically important as it allowed patients who had stopped taking metformin because of digestive problems to be able to resume metformin via the co-administration of the probiotics. 

Similarly, Chen et al. (2023) [20] demonstrated reduced HbA1c level along with homeostasis model assessment-β (HOMA-β). Chen et al. (2023) [20] also performed metagenomic and metabolomic analyses which showed increased abundance of gut SCFA-producing bacteria and bile acids. Significantly more bile acids and related metabolites were detected in their probiotic group than in the placebo group after intervention. These microbiome and metabolic shifts with probiotic use help to enhance the glycemic control. 

Taken together, the studies demonstrating positive therapeutic effects of probiotics on patients with T2DM on metformin is indicative that patients on metformin may present with a dysregulated gut microbiome. In fact, metformin has been associated with gastrointestinal side effects. Indeed, metformin use has been demonstrated to alter the gut microbiome composition in various rodent and clinical studies [64]. These microbiome changes negatively impact glycemic control since a healthy gut microbiota has been shown to provide glucose-lowering effects [65]. In summary, patients with T2DM taking metformin may benefit from the co-administration of probiotics for enhanced glycemic control by negating the gastrointestinal side effects. 

### 4.6. Limitations and Issues of the Studies

Unfortunately, many of the studies in this review lacked overall consistency in terms of biochemical parameters and data collection. For example, the studies in this review used varying primary and very diverse secondary outcomes. Moreover, anthropometric measurements were sometimes given for group baseline but not reported as deliverable outcomes. In addition, some studies acknowledged a parameter collection in their methods but did not provide a proper result, indicating reporting bias. Furthermore, some trials utilized questionable study designs. For example, Asemi et al. (2016) [45] had a 3-week washout period for their crossover study. Considering the modulating effects of probiotics, the 3-week period is likely inadequate for a proper washout. Also, the average length of probiotic administration in these articles was below 3 months, even though parameters like HbA1C describe a 3-month period. Additional issues were related to testing of multiple variables simultaneously. For example, Raygan et al. (2018) [40] and Asemi et al. (2016) [45] tested the probiotics with vitamin D and beta-carotene, respectively. Their control groups lacked these factors, so the stated probiotic results from these studies cannot be attributed to the probiotic use alone. Another issue is related to the inconsistency of the placebo. While some trials used basic placebos (e.g., sugar in capsules), it is important to consider that this is an unfavorable placebo for a CT involving patients with T2DM. Additionally, some studies listed specific medication in their inclusion or exclusion criteria, while this information was not provided in other studies. Based on the results presented with patients with T2DM and on metformin, it is of utmost importance that future studies provide detailed information regarding ongoing patient treatment, such as the medications and doses. 

We also noted that most studies had small sample numbers, and that this may potentially lead to bias which may influence some of the findings. In addition, the compliance variability to probiotic consumption as per protocol must be considered. For example, articles like Ahmadian et al. (2022) [24] stated compliance at 90% while others like Palacios et al. (2020) [19] set a threshold of 80%. The reporting of evidence is also difficult to interpret as studies use different statistical approach to support their hypothesis. The following statistical statements were reported: trending to significance, significantly different from baseline, significantly different between the groups, and significantly different between the groups upon addressing covariates. Each statistical approach may lead to a different interpretation of the data. As consideration for future trials, studies should use randomized groups compared directly by two-sample methods [66]. 

In general, the analysis results for the microbiome composition across the studies were difficult to interpret because of variations in study population, test product and outcome measures and the limitations of the study design [56]. For starters, studies using fermented products technically used probiotics as their placebo. Ismail et al. (2021) [30] saw their control group and interest group improve from baseline but were unable to state clinically significant results. In addition, ethnicity and geography drastically influence the microbiome. Moreover, Diabetes Canada has stated that ethnic background is a factor for developing T2DM. The studies in this review are from different parts of the world, where the varying environments may influence the microbiome and T2DM outcomes. While some studies collected certain demographics, the complexity of the microbiome cannot be understated, and its generalizability is always limited to the specific populations tested.

### 4.7. Next Steps and Recommendations

NGP (next-generation probiotics) are being studied as therapeutic agents that modulate the gut microbiota and disease progression. Some probiotics are being constructed to address hyperglycemia, offering a promising approach to T2DM treatment. Hu et al. (2023) [67], for example, constructed a novel and antidiabetic NGP that eases diabetic symptoms, modulates the microbiome, and reduces inflammation in pancreatic tissue in mice models. The future of these NGPs seem promising, but further CTs need to be completed in order to allow for this exciting path forward. 

As reported by Quigley and Gajula (2020) [56], “the goal of microbiome modulation is admirable, but we must clarify our objectives, fine-tune our interventions, and agree on outcomes” [56]. Upon analyzing the reviews in this study, the following recommendations are proposed for future CTs evaluating the use of probiotics in the management of T2DM (Figure 2):

**1.**  **Trial type and reporting:** Double-blind, randomized, placebo-controlled study with statistical significance considered between the groups is mandatory. It is important that trials have different participants in groups, as crossover designs and washout periods are not sufficient for microbiome studies. Trials should provide all probiotic information including the duration of probiotic administration, the CFU, the frequency, the mechanism/vector, the composition, the placebo substitute, and the specific probiotic strains. Pharmacotherapy should also be detailed. Studies need to report all data, even if it is through supplementary information. All parameters collected should be made accessible as selective reporting leads to bias.
**2.**  **Probiotic composition:** Multi-genus probiotics (≥two genera) rather than single-genus probiotics are predominantly used across the 33 clinical studies presented in this review. Based on the overall studies, we recommend probiotics consisting of *Lactobacillus* and *Bifidobacterium*, the most common combination of the genera studied across the studies (16 CTs).
**3.**  **Probiotic dose:** We recommend a probiotic dose of 4.0 × 10^10^ CFU/Day. This value is based on the median dose of studies with improvement in at least one glycemic parameter.
**4.**  **Use proper placebo:** The studies should utilize placebos, not just control groups. The placebos should be the modification of a single variable, the probiotic. Moreover, the placebo composition should be sugar-free as sugar affects T2DM and may interfere with the results. It may be prudent to use the same mechanism/vector as that of the probiotic but simply omit the probiotic aspect without noticeability.
**5.**  **Trial length:** We recommend a minimum of 3 months of probiotic administration to achieve clinically successful glycemic improvement with probiotic intervention for T2DM management. Rationale: ≥3 months of probiotic intervention resulted in higher frequency of studies (83%) demonstrating improved glycemic parameters compared to < 3 months of probiotic intervention (60%).
**6.**  **Assessment of biochemical parameters:** Studies need to collect and publish key biochemical parameters as outlined here: glycemic parameters (HbA1c, FPG, PPG, insulin, and HOMA-IR), lipid parameters (TC, HDL, LDL, VLDL, and TG), and blood pressure parameters (SBP and DBP). In addition, anthropometric measurements should be treated as outcomes, not just group baseline characteristics.
**7.**  **Significance:** Interpretation of clinically significant results should be performed using a between-group comparison and an analysis of respective differences from baseline. Studies should consider factoring covariates, if appropriate.
**8.**  **Removal of bias:** Multiple biases are identified throughout this review, with reporting bias being acknowledged the most. Future studies need to use standardized tools to limit bias.

## 5. Conclusions

Probiotics have been acclaimed for ages for a variety of health benefits; however, most are still awaiting evidence-based confirmation in high-quality CTs [56]. This review provides a summary of 33 CTs investigating the use of probiotic intervention for the management of T2DM (Figure 2). The lack of consistency with changes in the biochemical parameters with probiotic intervention may be attributed to differences in probiotic composition, duration of probiotic consumption, and probiotic dose. This emphasizes the need for additional research to address these knowledge gaps in terms of specific probiotic strains, dosages, and intervention plan. An interesting finding in this literature review was the beneficial trend of metformin and probiotic co-administration. Here, patients with T2DM taking metformin demonstrated enhanced glycemic control via the co-administration of probiotics. Taken together, the overall positive findings reported across the studies in combination with minimal adverse effects constitute ground for larger quality CTs to clearly identify the effects of probiotic use for the management of T2DM. This review provides recommendations that may address the shortcomings of the current studies and help to extract useful data from future CTs investigating the use of probiotics in T2DM management.

## Figures and Tables

**Table 2:** Probiotic characteristics.

Article ID	Probiotic Administration Characteristics									
Authors, Year of Publication	Time				Delivery Method		Probiotic			
	Duration of Administration (Months)	Average CFU/Dose	Frequency/Day (Assumed 1 If Not Indicated)	Average Total CFU /Day	Mechanism/ Vector	Composition	Placebo Substitute	Probiotic Genus	Number of Different Strains	Specific Probiotic Strains
[20]	3	3.00 × 10^10^	1	3.00 × 10^10^	Sachet	NS	Maltodextrin	*Lactobacillus, Bifidobacterium*	5	*L. casei Zhang, B. lactis V9, L. plantarum P-8, L. rhamnosus Probio-M9, and B. lactis Probio-M8* (Probio-X; Jinhua Yinhe Biotechnology Co., Ltd.; Beijing, China)
[21]	3	1.80 × 10^10^	2	3.60 × 10^10^	Capsules	370 mg of prebiotics and fillers such as inulin, microcrystalline cellulose, D-mannitol, and stearic acid	Lacking the bacterial consortium	*Lactobacillus, Bifidobacterium, Bacillus, Leuconostoc, Pediococcus*	8	*Ba. subtilis De111TM, B. bifidum, B. longum, L. paracasei, L. plantarum TBC0036, L. reuteri, Le. mesenteroides TBC0037, P. acidilactici* (BlisterPak Pro, LLC in Lafayette, CO, USA)
[22]	1.5	NS	1	NS	Capsule	Fructooligosaccharides as prebiotic	Starch	*Lactobacillus, Bifidobacterium, Streptococcus*	7	*L. rhamnosus, L. casei, L. bulgaricus, L. acidophilus, B. breve, B. longum, S. thermophilus* (FamiLact; Zist Takhmir Pharmaceutical Co., Tehran, Iran)
[23]	3	4.65 × 10^8^	2	9.30 × 10^8^	Yogurt	100 g yogurt	Lacking the probiotics	*Lactobacillus, Bifidobacterium*	2	*L. acidophilus, B. lactis*
[24]	1.5	2.40 × 10^11^	2	4.80 × 10^11^	Capsule	100 mg of fructo-oligosaccharide with lactose as carrier substances	Magnesium stearate	*Lactobacillus, Bifidobacterium, Streptococcus*	7	*L. acidophilus, L. casei, L. bulgaricus, L. rhamnosus, B. breve, B. longum, S. thermophilus* (Familact; Zist Takhmir Pharmaceutical Co., Tehran, Iran)
[25]	3	1.66 × 10^8^	1	1.66 × 10^8^	Capsule	NS	NA	*Lactobacillus* *Streptococcus* *Clostridium*	3	*S. Faecalis, C. butyricum, B. mesentricus*
[26]	2.5	NS	3	NA	Tablet	12 mg	NA	*Bifidobacterium*	1	*B. bifidum G9-1* (Biofermin; Taisho Pharmaceutical, Tokyo, Japan)
[27]	3	NS	2	NS	Capsule	NS	NS	NS	NS	NS
[28]	3	4.00 × 10^9^	1	4.00 × 10^9^	Liquid	NS	NA	*Bacillus* (spores)	1	*B. clause* spores (Enterogermina Plus; Sanofi, Paris, France)
[29]	3	Varied, NS	1/day first 2 weeks; 2 capsules/day rest of trial	Varied, NS	Capsule	NS	NA	*Bacillus* (spores)	5	*B. licheniformis, B. indicus, B. subtilis, B. clausii, B. coagulans*
[30]	4	NS	NS	NA	Yogurt	2 cups fortified yogurt	NA	*Bifidobacterium*	1	*B. animalis dn-173 010*
	4	NS	1	NA	Solid	1 teaspoonful of natural baking yeast	NA	*Saccharomyces*	1	*S. cerevisiae*
[31]	3	3.20 × 10^9^	1	3.20 × 10^9^	Capsule	NS	Starch (Tian San Qi Company, Xiamen, China)	*Lactobacillus, Bifidobacterium, Streptococcus*	3	*B. bifidum, L. acidophilus, S. thermophilus* (LactoCare, Zist Takhmir Pharmaceutical Co., Tehran, Iran)
[32]	6	9.00 × 10^8^	2	1.80 × 10^9^	Powder	3.0 g/day probiotic and 7.5 g/day galacto-oligosccharides	NA	*Lacticaseibacillus, Bifidobacterium*	2	*La. paracasei YIT 9029 (strain Shirota: LcS), B. breve YIT 12272 (BbrY)* (Yakult Honsha Co., Ltd., Tokyo, Japan)
[33]	3	5.00 × 10^10^	1	5.00 × 10^10^	Capsule	NS	10 mg/day corn starch	*Lactobacillus*	1	*L. paracasei HII01*
[19]	3	1.00 × 10^10^	2	2.00 × 10^10^	Capsule	Probiotics, 40 mg microcrystalline cellulose, 5 mg silica, and 10 mg magnesium stearate	200 mg microcrystalline cellulose, 10 mg silica, and 10 mg magnesium stearate per capsule	*Lactobacillus, Bifidobacterium, Streptococcus, Saccharomyces*	8	*L. plantarum Lp-115,L. bulgaricus Lb-64, L. gasseri Lg-36, B. breve Bb-03, B. animalis sbsp. lactis Bi-07, B. bifidum Bb-06, S. thermophilus St-21 and S. boulardii DBVPG 6763*
[34]	2	1.00 × 10^8^	1	1.00 × 10^8^	Capsule	Maltodextrin	Lacking the probiotics	*Lactobacillus*	*1*	*L. casei* (Chr. Hansen, Hoersholm, Denmark)
[35]	1	1.01 × 10^10^	1	1.01 × 10^10^	Yogurt	100 mL/day conventional yogurt (*L. bulgaricus, S. thermophilus*)	NA	*Lactobacillus, Bifidobacterium, Streptococcus*	2	*L. acidophilus La-5, B. lactis BB-12*
[36]	3	3.00 × 10^10^	2	6.00 × 10^10^	Capsule	100 mg fructo-oligosaccharide	Maltodextrin	*Lactobacillus, Bifidobacterium, Streptococcus, Bacillus*	6	*L. salivarius UBLS22, L. casei UBLC42, L. plantarum UBLP40, L. acidophilus UBLA34, B. breve UBBr01, B. coagulans Unique IS2* (Unique Biotech Limited, Kolthur, Hyderabad, India)
[37,38]	6	5.00 × 10^9^	2	1.00 × 10^10^	Sachet	2 g freeze-dried maize starch and maltodextrins	Lacking the probiotics	*Lactobacillus, Bifidobacterium*	*8*	*B. bifidum W23, B. lactis W52, L. acidophilus W37, L. brevis W63, L. casei W56, L. salivarius W24, L. lactis W19, L. lactis W58* (Winclove probiotics, Amsterdam, The Netherlands)
[39]	6	4.00 × 10^9^	1	4.00 × 10^9^	Capsule	NS	NS	*Lactobacillus*	*1*	*L. reuteri* (GenMont Biotech Inc., Tainan City, Taiwan)
	6	2.00 × 10^9^	1	2.00 × 10^9^	Capsule	NS	NS	*Lactobacillus*	*1*	*L. reuteri* (heat killed) (GenMont Biotech Inc., Tainan City, Taiwan)
[40]	3	8.00 × 10^9^	1	8.00 × 10^9^	Capsule	NA	Starch (Barij Essence Pharmaceutical Company, Kashan, Iran)	*Lactobacillus, Bifidobacterium*	*4*	*L. acidophilus, B. bifidum, L. reuteri, L. fermentum* (Lactocare, Zist Takhmir Pharmaceutical Co., Tehran, Iran)
[41]	2	2.00 × 10^9^	1	2.00 × 10^9^	Milk	Soy milk	NA	*Lactobacillus*	*1*	*L. plantarum A7*
[42]	3	3.00 × 10^10^	2	6.00 × 10^10^	Sachet	NS	NS	*Lactobacillus, Bifidobacterium*	6	*L. acidophilus, L. casei, L. lactis, B. bifidum, B. longum, B. infantis* (Hexbio^®^ B-Crobes Laboratory Sdn. Bhd. (Ipoh, Malaysia)
[43]	4	4.00 × 10^10^	1	4.00 × 10^10^	Milk	Yakult 400LT (Yakult Honsha Co., Ltd., Tokyo, Japan)	NA	*Lactobacillus*	*1*	*L. casei*
[44]	1.5	2.00 × 10^9^	1	2.00 × 10^9^	Milk	120 g/d of conventional fermented goat milk (Embrapa Goat and Sheep, Ceara, Brazil)	*Streptococcus thermophilus TA-40 (Danisco, Sassenage, France)*	*Lacticaseibacillus, Bifidobacterium*	2	*L. acidophilus La-5, B. animalis subsp. lactis BB-12* (Chr. Hansen, Hoersholm,, Denmark)
[45]	1.5	9.00 × 10^7^	3	2.70 × 10^8^	Food	Probiotic, 0.1 g inulin (HPX) as prebiotic, 0.05 g beta-carotene with 0.38 g isomalt, 0.36 g sorbitol, and 0.05 g stevia per 1 g	Same substance without probiotic, inulin and beta-carotene	*Lactobacillus*	1	*L. sporogenes*
[46]	2	4.00 × 10^9^	3	1.20 × 10^10^	Bread	Bread 120 g/day (Probiotic + 0.07 g inulin per 1 g) (Sahar Bread Company, Tehran, Iran)	Lacking the probiotics and inulin	*Lactobacillus*	1	*L. sporogenes* (heat-resistant) (Tak Gen Zist Company, Tehran, Iran)
[47]	2	NS	1	NS	Tablet	Fructooligosaccharides	Farina (Pharmaceutics Department of Mashhad School of Pharmacy)	*Lactobacillus*	*1*	*L. coagulans* (Bioplus Company, Bangalore, India)
[48]	1	5.00 × 10^10^	2	1.00 × 10^11^	Milk	Starter culture (*Streptococcus thermophilus* and *Lactobacillus delbrueckii* spp. Bulgaricus) 11% skim milk powder, flavoring, agar, and sucralose	Lacking the probiotics	*Lactobacillus*	1	*L. gasseri* SBT2055 (LG2055)
[49]	2	1.11 × 10^9^	1	1.11 × 10^9^	Yogurt	Conventional yogurt (*L. bulgaricus, S. thermophilus*)	N/A	*Lactobacillus, Bifidobacterium*	2	*L. bulgaricus*, *S. thermophiles*
[50]	2	4.00 × 10^9^	3	1.20 × 10^10^	Bread	120 g/day (Probiotic) (Sahar Bread Company, Tehran, Iran)	Lacking the probiotics	*Lactobacillus*	1	*L. sporogenes* (heat-resistant) (Tak Gen Zist Company, Tehran, Iran)
[51,52]	1.5	7.74 × 10^6^	1	7.74 × 10^6^	Yogurt	Conventional yogurt (*L. bulgaricus, S. thermophilus*) (Iran Dairy Industries Co., Tehran, Iran)	N/A	*Lactobacillus, Bifidobacterium*	2	B. lactis Bb12, L. acidophilus La5 (Chr. Hansen, Hoersholm, Denmark)
[53]	1	8.00 × 10^8^	2	1.60 × 10^9^	Shake	Probiotics, 9% skim milk powder, 23% whey powder, 21% maltodextrin, 15% oatmeal, 7% texturized soy-bean protein TSP, 5% soybean fiber, 3.5% guar gum, 3.5% collagen, 5% soybean extract, 4.5% fructooligosaccharide, and other	Lacking the probiotics and fructooligosaccharide.	*Lactobacillus, Bifidobacterium*	2	*L. acidophillus, B. bifidum*

**Abbreviations: ***B.* (*Bifidobacterium*), *L.* (*Lactobacillus*), N/A (not applicable), NS (not specified), and S. (*Streptococcus*).

**Table 3:** Summary of biochemical outcomes for studies with probiotics administration for ≥ 3 months.

Study	Probiotic Composition (Prebiotics, Genus)	Probiotic Duration	Glycemic Parameters					Lipid Parameters					Blood Pressure	
			HbA1c	FPG	PPG	Insulin	HOMA-IR	TC	HDL	LDL	VLDL	TG	SBP	DBP
[20] (T2DM patients on metformin)	*Lactobacillus, Bifidobacterium*	3 months	D	N.S.	N.S.	N.S.	N.S.	N.S.	N.S.	N.S.	N.T.	N.S.	N.T.	N.T.
[27] (T2DM patients on metformin)	*Not specified*	3 months	D	N.S.	N.S.	N.T.	N.T.	N.S.	N.S.	N.S.	N.T.	N.S.	N.T.	N.T.
[25] (T2DM patients on metformin)	*Lactobacillus, Streptococcus, Clostridium*	3 months	D	D	D	N.T.	N.T.	D	N.S.	D	N.T.	D	N.T.	N.T.
[36] (T2DM patients on metformin)	*Lactobacillus, Bifidobacterium, Streptococcus, Bacillus*	3 months	D	N.S.	N.T.	N.S.	N.S.	N.S.	N.S.	N.S.	N.T.	N.S.	N.T.	N.T.
[19] (T2DM patients on metformin)	*Lactobacillus, Bifidobacterium, Streptococcus, Saccharomyces*	3 months	D	D	N.T.	D	N.S.	N.S.	N.S.	N.S.	N.T.	N.S.	N.T.	N.T.
[42]	*Lactobacillus, Bifidobacterium*	3 months	D	N.S.	N.T.	D	N.S.	N.S.	N.S.	N.S.	N.T.	N.S.	N.S.	N.S.
[23]	*Lactobacillus, Bifidobacterium*	3 months	D	N.S.	N.S.	N.S.	N.S.	D	N.S.	D	N.T.	N.S.	N.T.	N.T.
[31]	*Lactobacillus, Bifidobacterium, Streptococcus*	3 months	D	D	N.S.	N.S.	N.S.	N.T.	N.T.	N.T.	N.T.	N.T.	N.T.	N.T.
[21]	*Lactobacillus, Bifidobacterium, Bacillus, Leuconostoc, Pediococcus*	3 months	N.S.	N.S.	N.S.	D	N.S.	N.S.	N.S.	N.S.	N.T.	N.S.	N.T.	N.T.
[28]	*Bacillus*	3 months	N.T.	D	N.T.	N.T.	N.T.	N.S.	I	N.S.	N.S.	N.S.	N.S.	N.S.
[33]	*Lactobacillus*	3 months	N.S.	D	N.T.	N.T.	N.T.	N.S.	I	D	N.T.	N.S.	N.T.	N.T.
[29]	*Bacillus*	3 months	N.S.	N.S.	N.T.	N.T.	N.T.	N.S.	N.S.	N.S.	N.T.	N.S.	N.S.	N.S.
[40]	Vitamin D, *Lactobacillus, Bifidobacterium*	3 months	N.T.	N.S.	N.T.	D	D	N.S.	I	N.S.	N.S.	N.S.	N.S.	N.S.
[30]	*Bifidobacterium*	4 months	D	D	N.S.	N.T.	N.T.	N.S.	N.S.	N.S.	N.T.	N.S.	N.T.	N.T.
[44]	*Lactobacillus*	4 months	N.S.	N.S.	N.T.	N.T.	N.T.	N.S.	N.S.	N.S.	N.T.	N.S.	N.T.	N.T.
[37,38]	*Lactobacillus, Bifidobacterium*	6 months	N.T.	D	N.T.	D	D	D	N.S.	N.S.	N.T.	D	N.T.	N.T.
[32]	*Lacticaseibacillus, Bifidobacterium*	6 months	N.S.	N.S.	N.T.	N.T.	N.T.	N.S.	N.S.	N.T.	N.T.	N.S.	N.T.	N.T.
[39]	*Lactobacillus*	6 months	D	N.S.	N.T.	N.S.	N.T.	D	N.S.	N.S.	N.T.	N.S.	D	D

Abbreviations: D (significant decrease in probiotic treatment group compared to placebo control; green box), I (significant increase in probiotic treatment group compared to placebo control; green box), N.S. (non-significant difference in probiotic treatment group compared to placebo control; beige box), and N.T. (parameter not tested; grey box).

**Table 4:** Summary of biochemical outcomes for studies with probiotics administration for < 3 Months.

Study	Probiotic Composition (Prebiotics, Genus)	Probiotic Duration	Glycemic Parameters					Lipid Parameters					Blood Pressure	
			HbA1c	FPG	PPG	Insulin	HOMA-IR	TC	HDL	LDL	VLDL	TG	SBP	DBP
[26]	*Bifidobacterium*	2.5 months	N.S.	N.S.	N.T.	N.T.	N.T.	N.T.	N.T.	N.T.	N.T.	N.T.	N.T.	N.T.
[34]	*Lactobacillus*	2 months	N.S.	D	N.T.	D	D	N.T.	N.T.	N.T.	N.T.	N.T.	N.T.	N.T.
[24]	Fructooligosaccharides, *Lactobacillus, Bifidobacterium, Streptococcus*	1.5 months	N.T.	N.T.	N.T.	N.T.	N.T.	N.S.	N.S.	N.S.	N.T.	N.S.	D	D
[47]	Fructooligosaccharides, *Lactobacillus*	2 months	N.T.	N.T.	N.T.	N.T.	N.T.	D	N.S.	N.S.	N.T.	N.S.	N.T.	N.T.
[22]	Fructooligosaccharides, *Lactobacillus, Bifidobacterium, Streptococcus*	1.5 months	N.T.	N.S.	N.T.	D	N.S.	N.S.	I	I	N.T.	N.S.	D	D
[53]	Fructooligosaccharides, *Lactobacillus, Bifidobacterium*	1 month	N.T.	D	N.T.	N.T.	N.T.	N.S.	I	N.S.	N.S.	N.S.	N.T.	N.T.
[51,52]	*Lactobacillus, Bifidobacterium*	1.5 months	D	D	N.T.	N.S.	N.T.	N.T.	N.T.	N.T.	N.T.	N.T.	N.T.	N.T.
[35]	*Lactobacillus, Bifidobacterium, Streptococcus*	1 month	N.T.	N.S.	N.T.	N.T.	N.T.	N.S.	I	N.S.	N.T.	N.S.	N.T.	N.T.
[50]	*Lactobacillus, Bifidobacterium*	2 months	N.T.	N.T.	N.T.	N.T.	N.T.	N.S.	I	D	N.T.	N.S.	N.T.	N.T.
[48]	*Lactobacillus*	1 month	N.S.	N.S.	N.T.	N.S.	N.T.	N.S.	N.S.	N.S.	N.T.	D	N.T.	N.T.
[41]	*Lactobacillus*	2 months	N.T.	N.S.	N.T.	N.T.	N.T.	N.T.	I	D	N.T.	N.S.	N.T.	N.T.
[43]	*Lacticaseibacillus, Bifidobacterium*	1.5 months	D	N.S.	N.T.	N.S.	N.S.	D	N.S.	D	N.T.	N.S.	N.T.	N.T.
[46]	*Lactobacillus*	2 months	N.T.	N.T.	N.T.	N.T.	N.T.	N.T.	N.T.	N.T.	N.T.	N.T.	N.S.	N.S.
[49]	*Lactobacillus*	2 months	N.T.	N.T.	N.T.	N.T.	N.T.	N.S.	N.S.	N.S.	N.S.	N.S.	N.T.	N.T.
[45]	Inulin, β-carotene, *Lactobacillus*	1.5 months	N.T.	N.S.	N.T.	D	D	D	N.S.	N.S.	D	D	N.S.	N.S.

Abbreviations: HbA1c (glycosylated hemoglobin A1C), FPG (fasting plasma glucose), PPG (postprandial glucose), HOMA-IR (homeostatic model assessment for insulin resistance), TC (total cholesterol), HDL (high-density lipoprotein), LDL (low-density lipoprotein), VLDL (very low-density lipoprotein), TG (triglycerides), SBP (systolic blood pressure), DBP (diastolic blood pressure), D (significant decrease in probiotic treatment group compared to placebo control; green box), I (significant increase in probiotic treatment group compared to placebo control; green box), N.S. (non-significant difference in probiotic treatment group compared to placebo control; beige box), and N.T. (parameter not tested; grey box).

## Data Availability

Not applicable.

## Abbreviations

BMI	body mass index
CFU	colony forming units
CTs	clinical trials
DBP	diastolic blood pressure
FPG	fasting plasma glucose
GI	gastrointestinal
HbA1c	glycosylated hemoglobin A1C
HDL	high-density lipoprotein
HOMA-IR	homeostatic model assessment for insulin resistance
LDL	low-density lipoprotein
NGP	next-generation probiotics
PPG	postprandial glucose
RCT	randomized control trial
SBP	systolic blood pressure
SCFA	short-chain fatty acids
TC	total cholesterol
TG	triglycerides
T2DM	type 2 diabetes mellitus
WHR	waist-to-hip ratio
